# Assessment of Learning Styles and Reaction Times Among Health Care Students: A Cross-Sectional Study

**DOI:** 10.7759/cureus.110492

**Published:** 2026-06-08

**Authors:** Ramalakshmi S, Abirami Omprakash

**Affiliations:** 1 Physiology, Sri Ramachandra Institute of Higher Education and Research, Chennai, IND

**Keywords:** auditory reaction time, health care students, kinesthetic, learning style preferences, learning styles, multimodal, psychomotor performance, unimodal, vark questionnaire, visual reaction time

## Abstract

Introduction

Reaction time is a simple objective indicator of central nervous system information-processing efficiency and reflects attention, sensory, and motor responses. The variation in reaction time among learning style preferences among health care students remains inadequately explored.

Objective

The objective of this study is to determine the distribution of VARK learning-style preferences among health care students and to evaluate the differences in visual and auditory reaction time across the various learning-style groups.

Methods

This single-center analytical cross-sectional study enrolled 120 health care students (B. Pharm) through convenience sampling at Sri Ramachandra Medical College and Research Institute. Learning styles were assessed using the VARK questionnaire, and visual and auditory reaction times were recorded. Statistical analysis was performed to evaluate the association between learning styles and reaction time.

Results

Among 120 health care students, the mean ± SD age was 22.52 ± 5.63 years, with females constituting 63/120 (52.5%) participants and males constituting 57/120 (47.5%) participants. Multimodal learning preference was most common among 62/120 (51.7%) students, followed by aural preference in 35/120 (29.2%) students and kinesthetic preference in 14/120 (11.7%) students, while visual preference was least frequent, in 9/120 (7.5%) students. Overall mean ± SD auditory reaction time (ART) was 151.65 (7.55) ms, and visual reaction time (VRT) was 199.85 (10.08) ms. ART differed significantly by learning style (p<0.001), with fastest responses in aural learners (146.11±4.62 ms) and slowest in kinesthetic learners (166.07±5.92 ms). VRT also varied significantly (p<0.001), being fastest in visual learners (184.22±2.59 ms) and slowest in kinesthetic learners (218.93±8.23 ms). ART and VRT showed a weak but significant positive correlation (r=0.299; p<0.001; R²=0.089).

Conclusion

Learning-style preferences were associated with significant differences in auditory and visual reaction times, with aural and visual learners showing faster modality-specific responses and kinesthetic learners demonstrating slower overall reaction times.

## Introduction

Health care students are trained for environments in which timely recognition of cues and rapid responses can influence patient outcomes, particularly during acute deterioration and other time-critical events where delays in action are associated with preventable harm [1]. In clinical teams, decision-making under time pressure is also regarded as a core component of safe practice, especially in high-acuity settings [2]. Against this background, objective neurocognitive measures that reflect information-processing efficiency, such as reaction time, have received attention as simple indicators of sensorimotor responsiveness and attentional readiness.

Reaction time is defined as the time interval between the presentation of a stimulus and the initiation of an appropriate voluntary motor response. It reflects the integrated functioning of stimulus detection, central processing involving attention, perception, response selection, as well as motor execution [3]. Reaction time is influenced by several biological and situational factors, including age and sex, practice effects, fatigue, and other variations in alertness, which can systematically alter response speed even in healthy young adults [4,5]. Reaction time also increases under higher cognitive workload, reflecting slower processing when attention and working-memory demands rise [6]. In student populations, auditory reaction time is commonly shorter than visual reaction time, consistent with modality-related differences in sensory processing and central integration. Alongside reaction time, learner characteristics, such as study preferences, are often explored using “learning style” models. The VARK framework categorizes preferences for information intake as Visual, Aural, Read/Write, and Kinesthetic, and it also recognizes that many learners report multimodal preferences (using more than one modality) [7]. Studies among medical students have reported substantial heterogeneity of VARK profiles, with multimodal preferences frequently observed in early undergraduate cohorts [8,9]. However, Pashler et al. (2008) have cautioned that evidence supporting the “meshing hypothesis” (better outcomes when instruction is matched to a learner’s declared style) is limited, and preference should not be equated with proven instructional effectiveness [10]. Given the importance of efficient sensory-motor responses in clinical learning environments and the widespread use of VARK to describe learner preferences, the present study aimed to describe the distribution of VARK learning-style preferences among health care students and to examine how visual and auditory reaction times varied across these preference groups.

## Materials and methods

This was a single-center, institution-based, analytical cross-sectional study conducted in the Department of Physiology, Sri Ramachandra Institute of Higher Education and Research, Porur, Chennai, Tamil Nadu, over a period of six months (June-November 2025). The study was approved by the Institutional Human Ethics Committee (IHEC) with reference number CSP-MED/25/JUN/116/113, dated 26/06/2025. The Participant Information Sheet (PIS) was made available in the local language for better comprehension by the participants, and its contents were verbally explained to ensure their understanding and satisfaction. Students from B. Pharm programs who were willing to participate and provided written informed consent were included. Students were excluded if they reported pre-existing neurological or psychological disorders (e.g., ADHD, epilepsy, anxiety disorders), had severe visual or hearing impairments, or had sleep deprivation.

Learning-style preferences were assessed using the self-administered VARK questionnaire version 8.2 (Visual, Aural, Read/Write, and Kinesthetic), developed by Fleming and Mills [5], © Copyright Version 8.02 (2023) held by VARK Learn Limited, New Zealand. Formal permission to use the VARK questionnaire for the present study was obtained from the original copyright holders prior to data collection.

The sample size was estimated using data from a previous study by Kamal et al. (2021) [11]. which reported an 89% proportion related to reaction time and memory among paramedical students with different learning styles. Using a relative precision of 5% and a 95% confidence level, the calculated minimum sample size was 118, which was rounded up to 120 participants. Eligible health care students were recruited using convenience sampling. Baseline demographic details (name/identification, age, sex, and course of study) were recorded using a structured proforma.

The VARK model categorizes learning preferences into four domains: visual learners prefer diagrams, flowcharts, and graphical representations; aural learners prefer listening and verbal discussion; read/write learners prefer textual information, such as reading and note-taking; and kinesthetic learners prefer hands-on activities, demonstrations, and experiential learning. The questionnaire uses scenario-based items in which respondents may select one or more options that best reflect their preferred way of receiving information. Responses were scored to obtain modality-specific totals, and participants were categorized according to their predominant preference(s) as unimodal (single dominant modality) or multimodal (two or more strong modalities), consistent with standard VARK interpretation [12].

Visual reaction time (VRT) and auditory reaction time (ART) were then measured using a reaction time apparatus. Reaction time was operationally defined as the time interval between the presentation of a sensory stimulus and the participant’s voluntary motor response [4]. Testing was performed in a quiet, adequately illuminated environment with the participant seated comfortably and instructed to respond as quickly as possible to the stimulus by pressing the response key; the apparatus recorded latency in milliseconds [13,14]. For VRT, the stimulus was a visual signal (e.g., a light/LED), and for ART, the stimulus was an auditory signal (e.g., tone/buzzer). Participants were given standardized instructions and a brief familiarization trial to minimize learning effects, after which multiple readings were obtained for each modality, and the mean of valid trials was used for analysis to improve reliability. Reaction time testing was considered an index of alertness and sensorimotor coordination in response to auditory and visual stimuli [4].

Statistical analysis

Statistical analysis was performed using IBM SPSS Statistics for Windows, Version 27.0 (IBM Corp., Armonk, NY, USA). Continuous variables were summarised as mean ± standard deviation, and categorical variables were presented as frequency and percentage. Normality of continuous data was assessed using the Shapiro-Wilk test and visual inspection of distribution plots. Differences in mean reaction times across learning style groups were evaluated using one-way analysis of variance (ANOVA). The association between auditory and visual reaction times was assessed using Pearson’s correlation coefficient (r), and simple linear regression was used to describe the relationship between the two measures. All statistical tests were two-tailed, and a p-value < 0.05 was considered statistically significant.

## Results

Among the 120 participants, the mean ± SD age was 22.52 ±5.63 years. Females constituted a slight majority (63, 52.5%) compared with males (57, 47.5%). The most common learning style preference was multimodal (62, 51.7%), followed by aural (35, 29.2%) and kinesthetic (14, 11.7%), while visual preference was least frequent (9, 7.5%). The auditory reaction time was 151.65± 7.55 ms, and the visual reaction time was 199.85±10.08 ms (Table 1). 

**Table 1:** Baseline demographic characteristics, learning-style preferences, and reaction-time measures among study participants (N=120) Continuous variables are presented as mean ± SD and categorical variables as n (%). Pearson’s correlation test was used to evaluate the association between auditory and visual reaction times.

Variable	Value
Age (years)	22.52 ± 5.63
Gender	N (%)
Male	57 (47.5%)
Female	63 (52.5%)
Learning-style preference	N (%)
Aural	35 (29.2%)
Kinesthetic	14 (11.7%)
Multimodal	62 (51.7%)
Visual	9 (7.5%)
Reaction time	Mean ± SD
Auditory reaction time (ms)	151.65 ± 7.55
Visual reaction time (ms)	199.85 ± 10.08
Correlation analysis between auditory and visual reaction times	Pearson’s correlation test: r = 0.299; p<0.001

Auditory reaction time differed significantly across learning style preferences (p < 0.001). Students with an aural preference had the fastest auditory reaction time at 146.11± 4.62 ms, followed by the multimodal group at 150.16 ±3.26) ms, while the kinesthetic group showed the slowest auditory reaction time at 166.07 ± 5.92 ms; the visual group had an intermediate value of 161.00 ± 2.24 ms (Table 2).

**Table 2:** Comparison of auditory reaction time across learning-style preference groups among health care students (N=120) Data are presented as mean ± SD. Statistical analysis was performed using one-way analysis of variance (ANOVA).

Learning-style preference	N	Auditory reaction time(ms) (Mean ± SD)	F value	P value
Aural	35	146.11 ± 4.62	101.54	<0.001*
Kinesthetic	14	166.07 ± 5.92		
Multimodal	62	150.16 ± 3.26		
Visual	9	161.00 ± 2.24		

Visual reaction time also varied significantly by learning style (p < 0.001), with the visual preference group demonstrating the fastest visual reaction time at 184.22± 2.59 ms and the multimodal group at 195.37± 3.86 ms, whereas the kinesthetic group had the slowest visual reaction time at 218.93± 8.23 ms; the aural group recorded 204.17± 5.60 ms (Table 3).

**Table 3:** Comparison of visual reaction time across learning-style preference groups among health care students (N=120) Data are presented as mean ± SD. Statistical analysis was performed using one-way analysis of variance (ANOVA).

Learning-style preference	N	Visual reaction time (ms) (Mean ± SD)	F value	P value
Aural	35	204.17 (5.60)	122.18	<0.001*
Kinesthetic	14	218.93 (8.23)		
Multimodal	62	195.37 (3.86)		
Visual	9	184.22 (2.59)		

**Figure 1:**
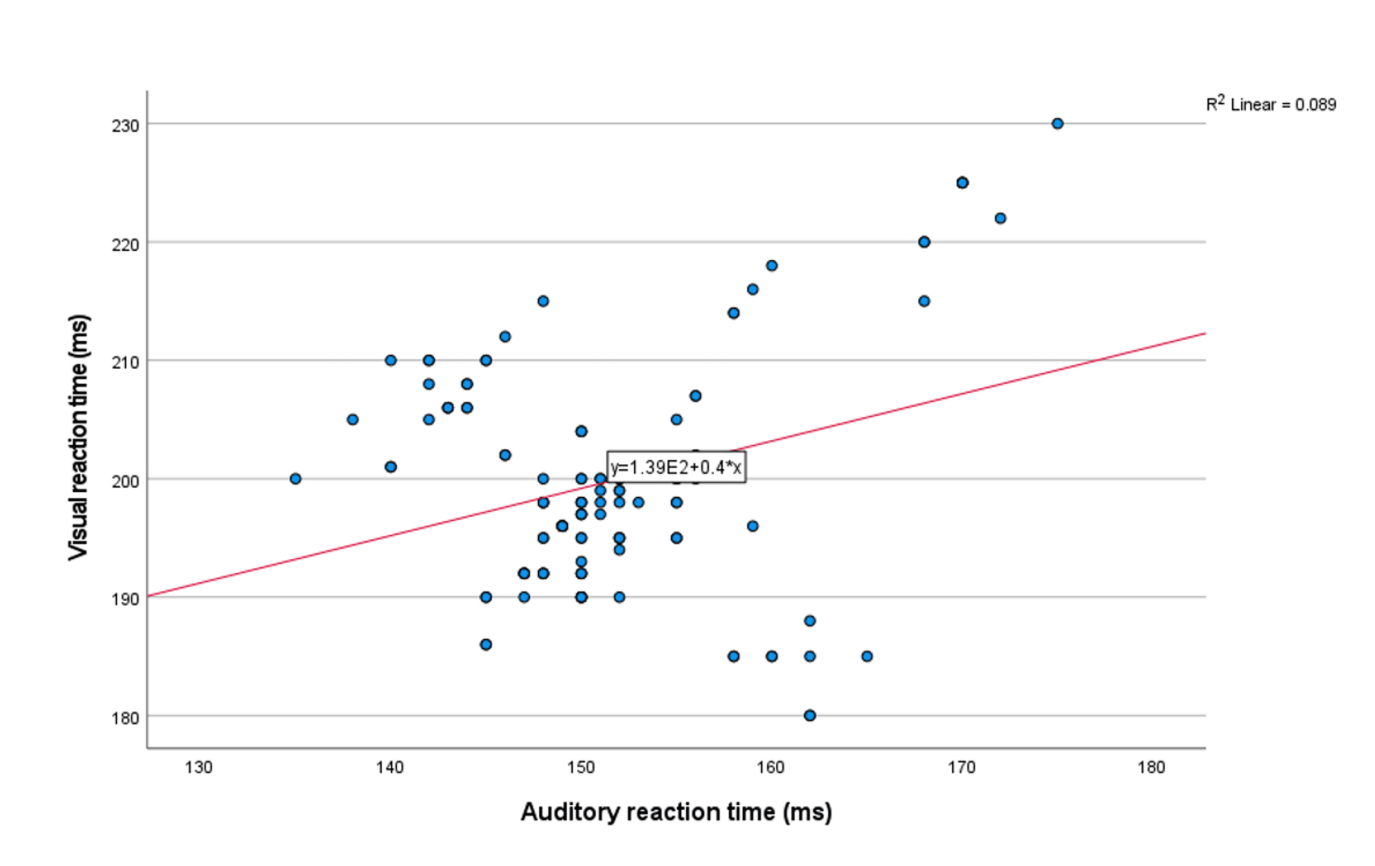
Scatter plot showing the correlation between auditory reaction time and visual reaction time among health care students (N=120) Pearson’s correlation test showed a weak positive correlation between auditory reaction time and visual reaction time (r = 0.298, p = 0.049), as added in the legend.

Pearson’s correlation analysis demonstrated a weak but statistically significant positive correlation between auditory and visual reaction times (r=0.298, p<0.001). The fitted linear model suggested a modest upward trend (approximately 0.4 ms increase in visual reaction time per 1 ms increase in auditory reaction time), with a low explained variance (R² = 0.089) (Figure 1).

## Discussion

The present findings describe learning-style preferences and sensory-motor processing speed in a cohort of young health care students (mean age 22.5 years), with a near-equal gender distribution. In cohorts of undergraduate health-professions learners, VARK-based surveys, including Fahim et al. (2021) and Urval et al. (2014), report a predominance of multimodal preferences, often interpreted as an inclination to combine more than one input channel (e.g., listening plus reading plus hands-on strategies) rather than relying on a single modality [12,15]. The VARK questionnaire itself conceptualizes preference across four modalities--Visual, Aural, Read/Write, and Kinesthetic--and permits selection of more than one option per item, which naturally facilitates identification of multimodal profiles when learners perceive multiple strategies as helpful [16]. In this context, the high proportion of multimodal preference (51.7%) observed in the present study is consistent with the way VARK is designed and with patterns reported in health-professions education settings, as noted by Aldosari et al. (2018) and Ojeh et al. (2017) [17,18].

Interpretation of VARK profiles warrants careful framing. “Learning styles” instruments are widely used to describe learners’ preferences, but Newton (2015) and Newton & Miah (2017) do not support the “meshing” or “matching” hypothesis-i.e., that instruction tailored to a declared learning style reliably improves learning outcomes [18,19]. Li et al. (2016) and Pashler et al. (2008) have highlighted psychometric and evidentiary limitations across learning-style models, and emphasize that preference should not be conflated with an empirically validated mechanism for optimizing achievement through matched teaching [20]. Nevertheless, learner preferences may still have practical value as a metacognitive prompt-encouraging students to diversify study strategies and to engage actively with material-provided they are not used deterministically or as a basis for restricting instructional design [21]. From an interprofessional education standpoint, the present distribution (multimodal > aural > kinesthetic > visual) suggests that many students were receptive to mixed-format learning environments that integrate listening/discussion with demonstrations, practice, and complementary visual aids, rather than relying on a single channel, in corroboration with Buşan (2014) and Hernandez et al. (2020) [22,23].

Reaction time outcomes add a neurophysiological dimension to these preference patterns. Reaction time is classically defined as the interval between stimulus presentation and initiation of the appropriate voluntary response, reflecting a composite of sensory transduction, central processing (attention, perception, decision/response selection), and motor execution [4]. In the present cohort, mean ART was 152 ms and mean VRT was 200 ms, showing the expected modality gap where responses to auditory signals are faster than to visual signals. A consistent physiological explanation is that auditory signals reach and engage cortical processing more rapidly than visual signals because of differences in receptor transduction and early pathway processing demands. Jain et al. (2015) and Mali et al. (2018) noted that auditory input can reach the brain in 8-10 ms, whereas visual input may take 20-40 ms, contributing to shorter ART compared with VRT [4,24], Hülsdünker et al. (2021) similarly reported that auditory reactions are typically 20-40 ms faster than visual reactions across simple tasks, reinforcing the plausibility of the ART-VRT separation seen here [25]. While absolute values vary by apparatus, stimulus properties, and task structure, the directionality (ART faster than VRT) is stable across laboratory paradigms, in corroboration with Jayaswal (2016) [26].

A key observation in this study was that both ART and VRT differed significantly across learning-style preference groups (p<0.001 for each). Students with an aural preference showed the fastest ART (146 ms), whereas kinesthetic-preferring students showed the slowest ART (166 ms), with multimodal and visual groups having intermediate ART. For VRT, the visual group was fastest (184 ms), multimodal was next (195 ms), aural was slower (204 ms), and kinesthetic was slowest (219 ms). These patterns are notable because they show modality-congruent performance at the group level: learners reporting an aural preference demonstrated the most efficient response to auditory stimuli, and learners reporting a visual preference demonstrated the most efficient response to visual stimuli. Such congruence is compatible with the idea that repeated engagement with a favoured channel (e.g., discussion/listening for aural; diagrams/graphs for visual) may be associated with faster orienting and response readiness to that stimulus type, through attentional biasing or greater familiarity with the sensory input [4]. However, it is also important to interpret these findings as associations rather than evidence that “teaching to a style” improves learning, given that reaction time reflects perceptual-motor speed under constrained conditions, not educational attainment or conceptual understanding. In other words, the present results may indicate that preference categories correlate with certain sensory-motor response tendencies, without implying that instructional matching would yield superior academic outcomes, as noted by Aboregela (2023) and Sha and Chiu (2025) [27,28].

The consistently slower ART and VRT in the kinesthetic group invites additional consideration. Kinesthetic preference in VARK is often described as favoring learning through experience, practice, and real or simulated activity rather than primarily through listening or visual symbolic representation. A plausible interpretation is that a simple button-press reaction task, especially if it is brief, decontextualized, and stimulus-driven, may align less closely with the kind of sustained sensorimotor integration and contextual feedback that kinesthetic learners prefer, potentially reducing engagement or anticipatory attention during the task [29]. Alternatively, kinesthetic categorization may be capturing broader individual differences (e.g., attentional control, arousal regulation, or comfort with highly constrained tasks) rather than a pure sensory modality effect, which could manifest as globally slower responses to both auditory and visual cues [30]. Since reaction time is sensitive to alertness and transient state factors, interpretation of between-group differences also benefits from acknowledging the role of standardization procedures and exclusions (e.g., sleep deprivation and caffeine) that reduce, but do not fully eliminate, variability in vigilance. Short-term sleep deprivation has been shown to impair cognitive performance and prolong reaction times [31], consistent with the rationale for excluding sleep-deprived participants from testing sessions. Caffeine, conversely, can alter attention and arousal and has demonstrated measurable effects on reaction time in experimental settings [32], supporting exclusion of recent caffeine intake to minimise acute stimulatory confounding.

The study also found a weak but statistically significant positive correlation between ART and VRT (r=0.299; p<0.001) with low explained variance (R²=0.089). This direction of association is expected because sensory reaction time across modalities typically shares common central components, such as general processing speed, sustained attention, and motor execution efficiency, despite modality-specific afferent pathway differences. Prior work by Jain et al. (2015) in a student population similarly examined correlations between auditory and visual reaction times, reinforcing that individuals who respond more slowly in one modality often tend to respond more slowly in the other, albeit with modest effect sizes because modality-specific processing contributes additional variance [4]. The small R² is also important; it indicates that most of the variability in VRT was not explained by ART alone, which is consistent with Lazar et al. (2021) that visual responses depend more heavily on additional stages such as visual feature encoding, spatial attention, and higher-order perceptual integration [33]. From an educational and skills-training perspective, reaction time measures offer a compact, objective index of alertness and sensorimotor responsiveness relevant to clinical tasks (e.g., responding to alarms, interpreting rapid visual cues, and executing timely actions) [25]. The observation that multimodal learners had relatively efficient performance for both ART and VRT (second-fastest in each modality) is consistent with the notion that flexible strategy use may co-occur with broader cognitive adaptability-although, again, the evidence does not support assuming that multimodality implies superior academic learning per se. Practically, these findings can still inform learner counselling: students may be encouraged to diversify study techniques (e.g., pairing diagrams with verbal rehearsal and practice-based application) and to adopt evidence-based learning strategies (retrieval practice, spaced repetition, elaboration) rather than relying solely on a declared style label [19].

The present study had certain limitations. As an analytical cross-sectional study, it captured learning-style preferences and reaction times at a single point in time and therefore could not establish causal relationships or determine whether learning preferences influenced reaction-time performance or vice versa. The study was conducted at a single institution with a modest sample size and included only selected health-care programs, which may limit generalizability to other colleges, disciplines, age groups, or cultural settings.

## Conclusions

An analytical cross-sectional study was conducted at a single center among 120 health care students. Multimodal learning preference was most common, followed by aural and kinesthetic preferences, with visual preference being least frequent. Auditory reaction time was faster than visual reaction time overall, consistent with expected sensory processing differences. Both auditory and visual reaction times differed significantly across learning style preference groups, with aural learners demonstrating the fastest auditory responses and visual learners demonstrating the fastest visual responses, while kinesthetic learners showed comparatively slower responses in both modalities. A weak but statistically significant positive correlation was observed between auditory and visual reaction times, indicating shared but limited variance in sensorimotor response speed across modalities. Overall, the findings suggested that learning-style preferences were associated with measurable differences in simple reaction-time performance, supporting the value of considering learner characteristics when planning multimodal instructional approaches and skill-based training in health-professions education.
